# TMEM150B is dispensable for oocyte maturation and female fertility in mouse

**DOI:** 10.1038/s41598-020-78554-1

**Published:** 2020-12-07

**Authors:** Ran Liu, Hanni Ke, Tong Shao, Yingying Qin, Shidou Zhao

**Affiliations:** 1 Center for Reproductive Medicine, Cheeloo College of Medicine, Shandong University, Jinan, 250012 Shandong China; 2 Key Laboratory of Reproductive Endocrinology of Ministry of Education, Shandong University, Jinan, 250012 Shandong China; 3 Shandong Key Laboratory of Reproductive Medicine, Jinan, 250012 Shandong China; 4 Shandong Provincial Clinical Research Center for Reproductive Health, Jinan, 250012 Shandong China; 5 National Research Center for Assisted Reproductive Technology and Reproductive Genetics, Shandong University, 44 Wenhua Xi Road, Jinan, 250012 Shandong China

**Keywords:** Animal physiology, Germline development

## Abstract

Premature ovarian insufficiency (POI) refers to severe decline of ovary function in females which usually leads to infertility. It has been reported that the *TMEM150B* gene is mostly associated with age at natural menopause, early menopause and POI, but its role in female reproduction remains unknown. In this study, we found *Tmem150b* was highly expressed in mouse oocytes, but its deletion had no obvious effect on meiotic maturation of oocytes indicated by first polar body emission and spindle morphology. There were also no obvious differences in follicle development and corpus luteum formation between knockout and wild type mice. Finally, knockout of *Tmem150b* did not affect female fertility and sexual hormone levels. In summary, our results suggest that TMEM150B is not essential for female fertility in mice.

## Introduction

Premature ovarian insufficiency (POI) causes fertility decline in women which is characterized by defect of ovarian function with serum follicle-stimulating hormone (FSH) level over 25 IU/L before the age of 40 years^1,2^. The prevalence of POI has been reported approximately to be 2.8% in Chinese women^3^. The etiology of POI is complex and highly heterogeneous. Different factors might be involved in POI including iatrogenic factors, genetic abnormalities, autoimmune disorders, idiopathic and environmental factors, while 20–25% of cases may be explained by genetic defects^4^. Causative genes associated with POI have been identified, such as genes associated with follicular development (*FIGLA*, *FSHR*, *NOBOX*, *GDF9* and *BMP15*), and genes related to meiosis and DNA repair (*STAG3*, *HFM1*, *MCM8*, *MCM9* and *MSH5*)^5^. However, the etiology of majority of POI cases still remains unclear.

Previous studies have shown that some genetic susceptible loci are shared by age at natural menopause (ANM), early menopause and POI^6^. Accordingly, several genes related to these loci, such as *MCM8, MSH5* and *SYCP2L*, have been identified^7,8^. Not only their knockout mice exhibit infertility, ovarian dysgenesis or premature insufficiency^9–11^, but also mutations in these genes were found in POI patients and the causality was confirmed by functional studies^12–15^. Besides, *TMEM150B* is one of the genes identified to be associated with ANM and early menopause, which is most significantly correlated with POI in human^16^. While TMEM150B was discovered to promote cell viability under glucose deprivation by prompting autophagy^17^, knockout of an essential autophagy-related gene *Atg7* in germ cells resulted in ovarian follicle loss and subfertility in female mice^18^. Whether TMEM150B is required for female fertility has not been investigated yet. Therefore, we generated the *Tmem150b* knockout mouse model to explore the potential role of TMEM150B in female reproduction.

## Materials and methods

### Quantitative reverse-transcription PCR (qRT-PCR)

To verify the expression of *Tmem150b* in oocytes and other tissues, qRT-PCR was performed with wild type mouse tissues. Total RNA was extracted according to the manufacturer’s protocol of Qiagen RNase mini kit (Invitrogen, USA). The cDNA was obtained by reverse transcription of RNA using PrimeScript reverse transcriptase (Takara, Japan). Real time PCR was performed using SYBR Green Master Mix (Takara, Japan) with specific primers. *Gapdh* was used as the internal control. Primer sequences are as follows: *Tmem150b*, F: 5′-TTGCTGCCTGTCATCTTATTTC-3′, R: 5′-AGGTTTTGACGCCCCAGT-3′; *Gapdh,* F: 5′-AGGTCGGTGTGAACGGATTTG-3′*,* R: 5′-TGTAGACCATGTAGTTGAGGTCA-3’.

### Establishment of *Tmem150b* knockout mice

*Tmem150b* knockout (KO) mice were constructed by CRISPR/Cas9 technique (Cyagen, Suzhou, China) with deletion of 989 bp fragment encompassing exons 2–4. Genotyping was performed by PCR from genomic DNA of mouse tails. Validation of gene knockout in mRNA level was done by RT-PCR using cDNA obtained by reverse transcription of mRNA from mouse ovaries and the PCR products were confirmed by Sanger sequencing. The sequences of primers were as follows: wild type (WT) allele, F: 5′-GACTGCTTGGAGATCCAGCT-3′, R: 5′-GTGGAGGCAGTCTGACTATC-3′; and delete allele, dF: 5′-CTTTGTGCCCTGGGTACCTC-3′, R: 5′-GTGGAGGCAGTCTGACTATC-3′; F’: 5′- AGTGGGACCACAAAAGCAGG-3′, R’: 5′- CCTTTCACCCACCAGGACAG-3′. All PCR products were separated by 1.5% (w/v) agarose gel, stained with ethidium bromide (Invitrogen, USA) and detected by Chemidoc MP System (Bio-Rad, USA). All genetically altered mice had a mixed background of ICR and C57BL/6J. WT mice for experiments were purchased from Charles Rive Company. Mice were housed in a temperature-controlled (22 ± 2 °C) room with a 12/12 h light/dark cycle and free access to water and food. All animal experiments were performed in accordance to the ethical guidelines approved by Animal Care and Research Committee of Shandong University.

### Collection of oocytes

For collection of fully-grown GV stage oocytes, 21-day-old female mice were injected with 5 IU PMSG (Ningbo Sansheng Pharmaceutical Corporation, Zhejiang, China) through intraperitoneal injection. After 46 h, the ovaries were minced with dissection blades before oocytes were collected after 3 times of wash. For MII oocytes collection, mice were super-stimulated with 5 IU PMSG for 46 h and MII stage oocytes were collected after 16 h of hCG (5 IU, Ningbo Sansheng Pharmaceutical Corporation, Zhejiang, China) administration by puncturing the oviduct under light microscopy.

### Immunofluorescence

Immunostaining of oocytes was performed as described previously^19^. Briefly, MII oocytes were fixed in 4% paraformaldehyde (pH = 7.4) for 30 min at room temperature, permeabilized in 0.3% TritonX-100 for 20 min, blocked with 1% BSA dissolved in PBS and then incubated with anti-α-Tubulin-FITC antibody for 1 h. DNA was counterstained with propidium iodide for 10 min at room temperature. Oocytes were mounted on glass slides after being washed three times with PBS and detected for fluorescence under high speed confocal laser scanning microscope (Dragonfly, Andor, England).

### Estrous cycle examination

Stages of estrous cycle were detected by preparing vaginal smears in early morning of 2-month-old mice for a period of 14 days. Cells were washed out of vagina with 20 μL PBS to glass slide and fixed in 95% methanol. Vaginal smears were examined after hematoxylin and eosin (H&E) staining and observed under light microscopy (BX53, Olympus, Japan).

### Determination of hormonal profile and histological analysis

To detect sex hormone levels from serum, blood was collected through ophthalmectomy immediately after the general anesthetic. According to standard protocol, serum samples were separated by centrifuging at 4 °C, 3000×*g* for 30 min. Follicle-stimulating hormone (FSH) and estradiol (E_2_) concentrations were measured by radioimmunoassay (Beijing North Institute of Biotechnology, China).

Ovaries from 3-month and 8-month-old mice were collected and fixed in Bouin’s solution at 4℃ overnight before being transferred to 70% (vol/vol) alcohol. Ovaries were processed, embedded in paraffin with standard protocols and serially sectioned at 5 μm. Following deparaffinization, ovarian sections were stained with H&E for further histological analysis.

### Fertility test

For fertility test, 2-month-old *Tmem150b*^+*/*+^ and *Tmem150b*^*-/-*^ females were mated with WT males over a period of 6 months. The number of pups was counted at the day of birth. Fertility was assessed by the total number of pups per genotype, average number of litters per mouse, average number of pups per litter and time to first litter.

### Statistical analysis

Data were presented as mean ± SD and analyzed by Student’s *t* test. Statistical analysis was performed with SPSS 16.0 software. Differences were considered to be statistically significant when *P* < 0.05.

## Results

### Oocyte meiotic maturation and spindle organization

**Figure 1:**
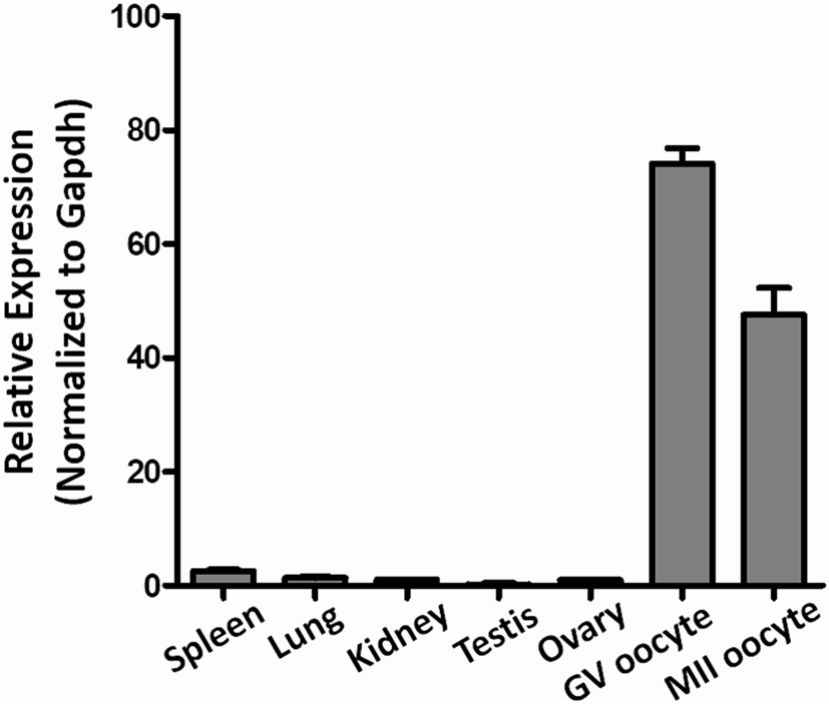
The mRNA expression of *Tmem150b* in mouse tissues and oocytes detected by qRT-PCR. The expression level of this gene in the ovary was set as “1” and other samples were normalized to it.

We firstly analyzed *Tmem150b* expression in a variety of mouse tissues by qRT-PCR. *Tmem150b* mRNA level was low in diverse mouse tissues containing spleen, lung, kidney, testis and ovary, while the expression of *Tmem150b* was relatively high in oocytes at GV and MII stages (Fig. 1). These results suggested that *Tmem150b* might play a role in mouse oocytes.

**Figure 2:**
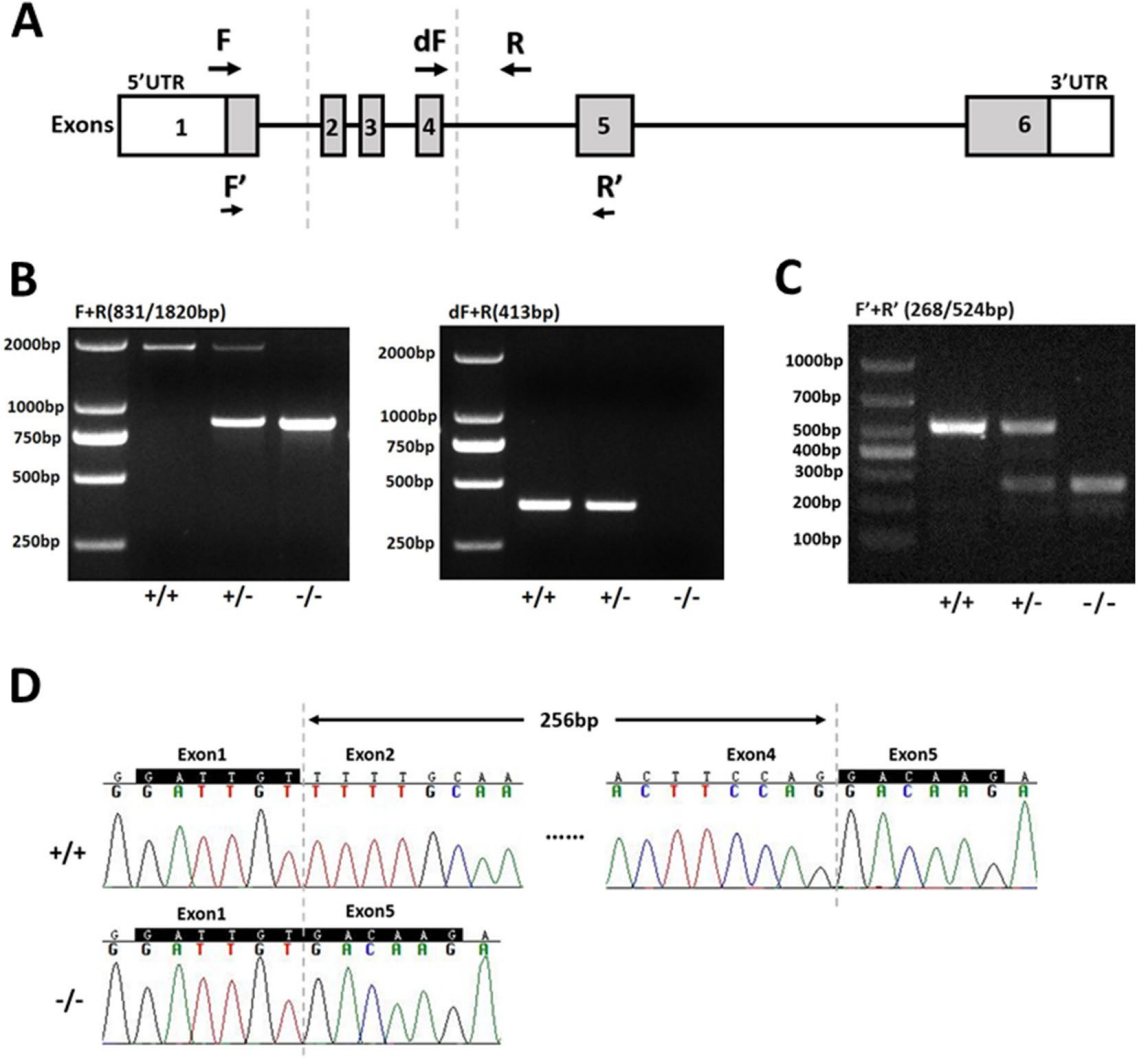
Targeted disruption of the *Tmem150b* in mouse genomic DNA and mRNA. **(A)** Schematic representation of the mouse *Tmem150b* gene. The exons and introns deleted are indicated by dashed line. Arrows show positions of PCR primers used for genotyping in genomic DNA and confirming the disruption of gene in mRNA level. **(B)** Genotyping of mice by PCR amplification. The PCR using primers (F + R) for wild type and knockout alleles yielded 1820 bp and 831 bp fragments respectively. The PCR using primers (dF + R) for wild type and knockout alleles yielded 413 bp and no bands respectively. **(C)** Confirming the knockout of *Tmem150b* in mRNA using RT-PCR with the primers (F’ + R’) for wild type and knockout alleles yielding 524 bp and 268 bp fragments, respectively. **(D)** Sanger sequencing showed the deletion of exons 2 to 4 in *Tmem150b* mRNA from *Tmem150b*^*-/-*^ mouse ovary.

**Figure 3:**
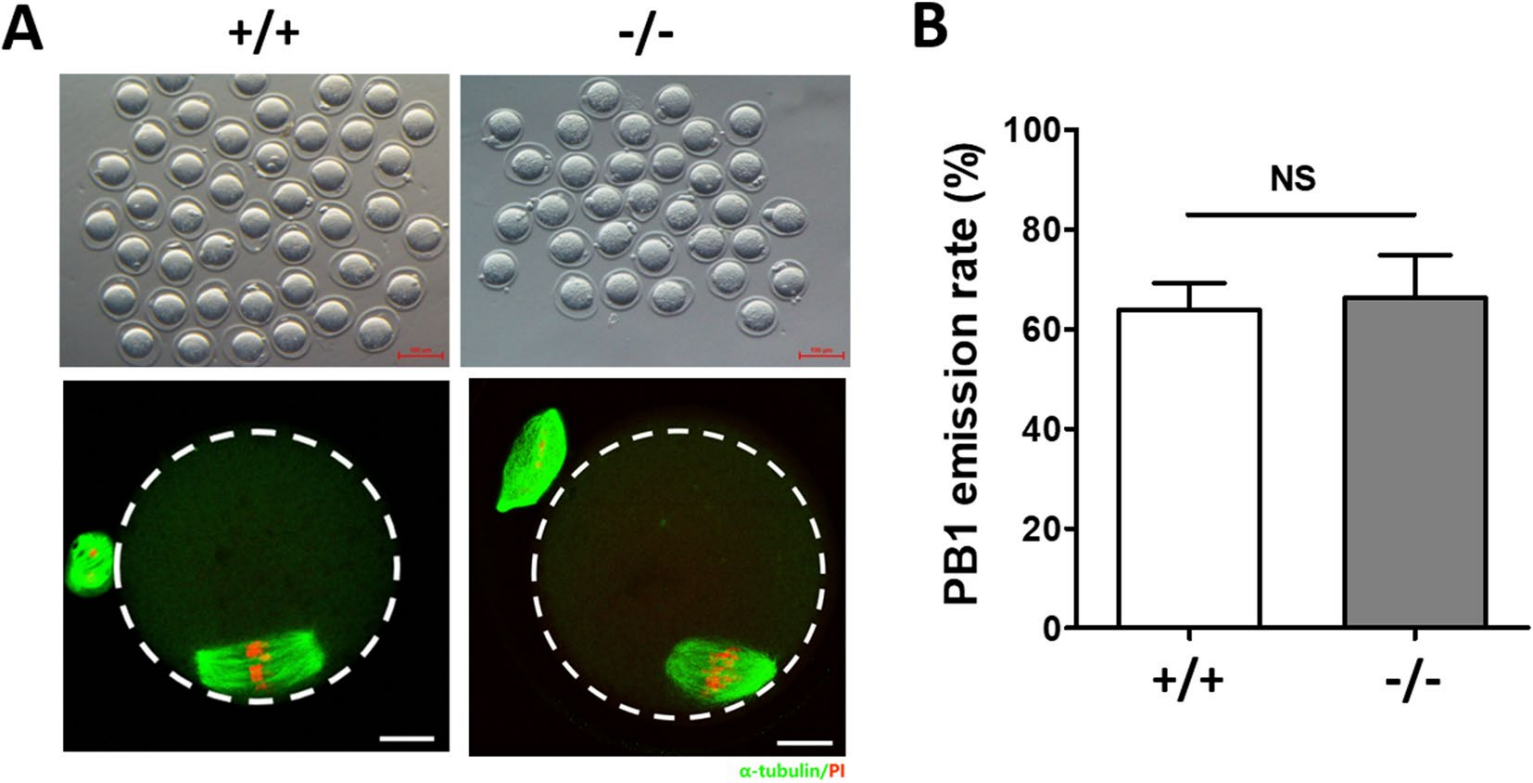
Oocyte maturation analysis by morphology and immunofluorescence staining. **(A)** Representative morphology of MII stage oocytes after superovulation at postnatal day 21 derived from *Tmem150b*^+*/*+^ and *Tmem150b*^*-/-*^ mice. Scale bar = 100 μm. The α-tubulin was stained by a fluorescein isothiocyanate (FITC)-conjugated antibody, and DNA by PI. Scale bar = 10 μm. **(B)** Statistical analysis of PB1 emission rates between *Tmem150b*^+*/*+^ (n = 236) and *Tmem150b*^*-/-*^ (n = 230) MII oocytes. NS, no significance.

To investigate the potential role of TMEM150B, we generated *Tmem150b* KO mouse model by deleting exons 2 to 4 (Fig. 2A). While PCR results using primers (F + R) for wild type allele yielded 1820 bp fragment, knockout allele produced 831 bp fragment. Meanwhile, PCR using another pair of primers (dF + R) yielded 413 bp and no bands for wild type and knockout alleles, respectively (Fig. 2B). We further carried out RT-PCR to validate the disruption of the expression of *Tmem150b* in ovaries from adult mice (Fig. 2C). While the WT allele yielded 524 bp band, KO allele produced 268 bp band. Sanger sequencing also confirmed the deletion of targeted exons 2 to 4 (Fig. 2D). There were no significant differences in body weight and body length between *Tmem150b*^+*/*+^ and *Tmem150b*^*−/−*^ mice according to phenotypical evaluation. MII stage oocytes were collected from WT and KO mice after overstimulation to determine the role of TMEM150B in oocyte meiotic maturation. Similar rates of first polar body (PB1) extrusion were observed in both genotypes (Fig. 3), suggesting that TMEM150B is dispensable for oocyte maturation.

Next, the spindle morphology of MII stage oocytes was observed from both groups. As shown by immunofluorescence staining, the spindle morphology from *Tmem150b*^*-/-*^ oocyte was similar to wild type oocyte (Fig. 3A). Furthermore, most metaphase oocytes presented typical fusiform-shaped spindles and well-aligned chromosomes at the equatorial plate, suggesting that absence of TMEM150B has no effect on spindle organization.

### Estrous cycle and follicular development of adult mice

**Figure 4:**
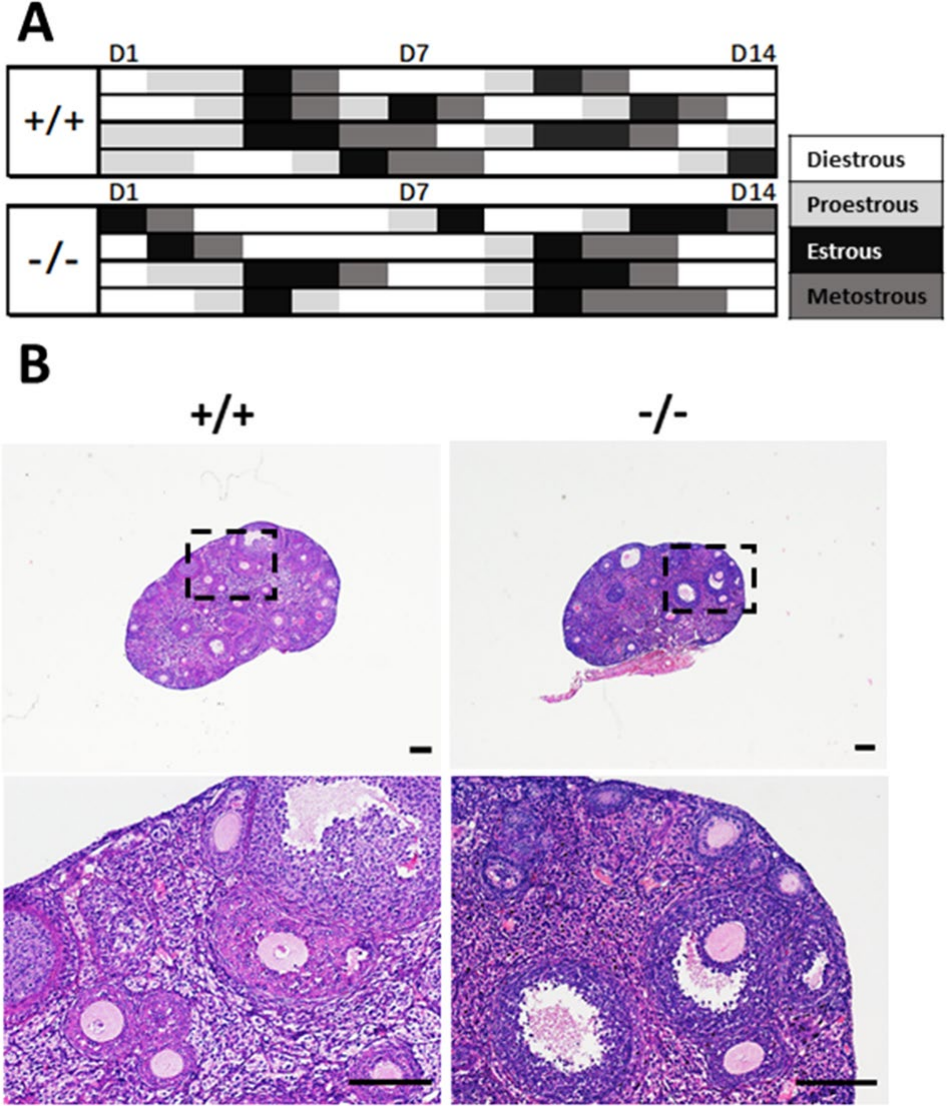
Estrous cycle analysis of female mice and morphology analysis of ovaries by H&E staining. **(A)** Regular estrous cycle of 2-month-old female mice from both genotypes. **(B)** Ovarian histological analysis of 3-month-old *Tmem150b*^+*/*+^ and *Tmem150b*^*-/-*^ females by H&E staining. Scale bar = 100 μm.

As there were no changes in oocyte maturation at puberty of mice, we wondered whether TMEM150B deletion had any effect on adult female mice. We examined the estrous cycle through vaginal smear with H&E staining every morning for 2 weeks. All the *Tmem150b*^*-/-*^ mice have regular estrous cycles and normal cell morphology compared with control mice, suggesting that loss of TMEM150B had no obvious effect on estrous cycle (Fig. 4A).

We also observed normal morphology of follicles at different developmental stages from both groups, including primordial follicles, primary follicles, secondary follicles, antral follicles and corpus luteum (Fig. 4B). Granulosa cells were also arranged regularly around oocytes. Collectively, these results suggest that *Tmem150b* is dispensable for folliculogenesis.

### Hormonal profile and female fertility

Fertility test was then performed by mating *Tmem150b*^+*/*+^ and *Tmem150b*^*-/-*^ females with WT males for a period of 6 months. Interestingly, all of the adult *Tmem150b*^*-/-*^ mice were showed normal fecundity during this time duration. As shown in Table1, the total number of pups in *Tmem150b*^*-/-*^ group was similar to that in control group. In addition, the average numbers of pups per mouse or per litter were also comparable between the two genotypes. Neither the giving birth at the first time showed any differences. All the newborn pups showed similar size between both genotypes.

**Table 1:** Fertility test results of two mouse genotypes.

Genotypes	Total pups	Litters/Mouse	Pups/Litter	Time to first litter (days)
+ / + (n = 5)	177	4.0 ± 0.45	8.85 ± 1.53	95 ± 3.98
−/− (n = 5)	189	4.2 ± 0.37	8.59 ± 0.82	98 ± 6.18

**Figure 5:**
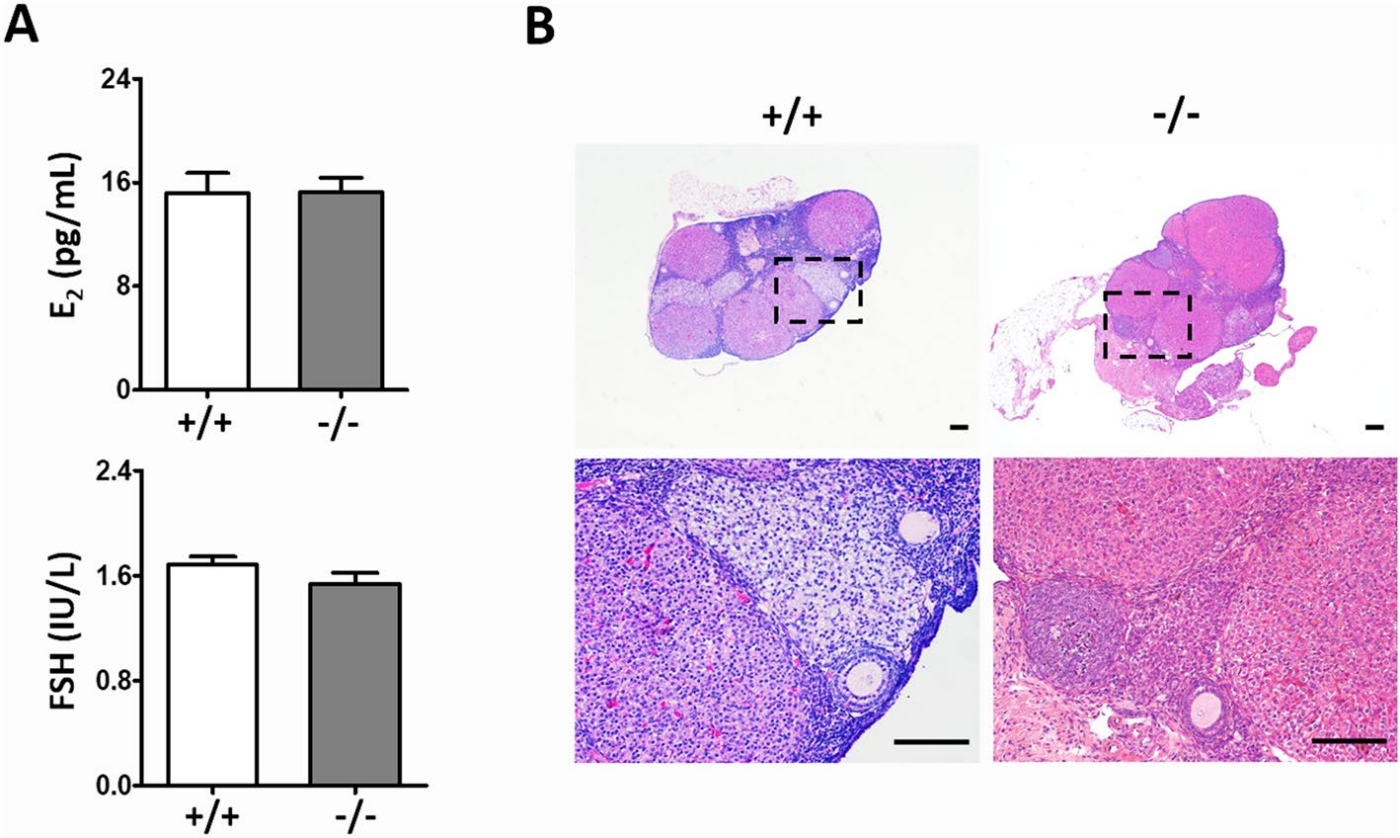
Hormone and ovary morphology analyses after fertility test. **(A)** No significant changes in FSH and E_2_ concentrations in serum of 8-month-old mice between *Tmem150b*^+*/*+^ (n = 5) and *Tmem150b*^*-/-*^ (n = 5) group. **(B)** Ovarian histological analysis with H&E staining after fertility test from both genotypes. Scale bar = 100 μm.

In addition, we determined whether *Tmem150b* deletion causes any change in hormonal profile and ovarian morphology in mice aged 8 months. The levels of FSH and E_2_ exhibited no obvious alterations between *Tmem150b*^+*/*+^ and *Tmem150b*^*-/-*^ mice (Fig. 5A). Finally, ovaries from both genotypes showed similar amount of follicles from different stages and corpus lutea (Fig. 5B), suggesting that the *Tmem150b*^*-/-*^ mice also own regular ovulatory ability.

## Discussion

Women suffering from POI face infertility with adverse health outcomes^1^. Despite the etiology of POI has been discovered over decades, the mechanisms still have not been fully investigated. It has been reported that early menopause shares a similar genetic pattern with idiopathic POI^20^. *TMEM150B* has been recognized as one of candidate genes which are significantly associated with ANM and early menopause^7,8^. Moreover, the SNP rs11668344 located in intron 2 of the *TMEM150B* gene has been verified to be most significantly associated with POI in European ancestry through genome-wide association study^16^. However, this variant was not verified and no mutation of *TMEM150B* was identified in the Chinese POI population in our previous study^21^.

It has been reported that the *TMEM150B* gene was expressed in human ovary^17^. In the present study, we further confirmed the high expression of *Tmem150b* in mouse oocytes in immature and mature stages. The deletion of genomic DNA from our gene-edited mice was large enough to truncate the TMEM150B protein and make it non-functional. Consequently, we found that the deletion of *Tmem150b* had no effect on follicle maturation and oocyte development, as indicated by similar PB1 extrusion rate and MII oocyte morphology. Furthermore, fertility test showed similar number of pups and litter size in both genotypes. Estrous cycle and hormonal profile also exhibited no significant differences between both genotypes. Our findings suggest that TMEM150B is dispensable for folliculogenesis, oocyte maturation and female fertility in mice.

Although our study demonstrates that *Tmem150b* does not play a prominent role in follicle development and fertility of female mice, there may be some limitations. TMEM150B, also named as DRAM-related/associated member 3 (DRAM-3), is one of the five members of the DRAM family which includes DRAM-1, DRAM-2 and TMEM150A/B/C^17,22^ and functional redundancy cannot be excluded. It may be better to mutate all interrelated genes to eliminate the compensation from paralogs. Besides, we only detected the phenotype at basal status in normal condition, and could not exclude the occurrence of POI phenotype under stress conditions.

In summary, we demonstrate that the female *Tmem150b*^*-/-*^ mice have normal fertility. Together with our previous study identifying no causative variants in POI patients, it is suggested that the *TMEM150B* gene may not be a POI causative gene. Nevertheless, our study may help the scientific community reveal the etiology of POI.
